# Hunter's Silicious Cement

**Published:** 1852-04

**Authors:** 


*Hunter's Silicious Cement.*—In a former number of the Journal, we took occasion to notice some specimens of artificial teeth sent to us for inspection by Dr. Hunter, of Cincinnati, Ohio, mounted on gold plates with a beautiful artificial gum formed of a silicious composition fused to the teeth and plates. Since then, a gentleman from the west, wearing a double set of artificial teeth mounted by Dr. H. in the same manner, called upon us, that we might have an opportunity of seeing them in the mouth. The teeth were mounted on gold plates, accurately adapted to the parts on which they rested, and were worn with great comfort and satisfaction, answering all the purposes which a substitute for the natural organs is capable of subserving.

